# Two New Cyathane Diterpenoids from Mycelial Cultures of the Medicinal Mushroom *Hericium erinaceus* and the Rare Species, *Hericium flagellum*

**DOI:** 10.3390/ijms19030740

**Published:** 2018-03-06

**Authors:** Zeljka Rupcic, Monique Rascher, Sae Kanaki, Reinhard W. Köster, Marc Stadler, Kathrin Wittstein

**Affiliations:** 1 Department Microbial Drugs, Helmholtz Centre for Infection Research GmbH, Inhoffenstraße 7, 38124 Braunschweig, Germany; zeljka.rupcic@helmholtz-hzi.de (Z.R.); monique.rascher@helmholtz-hzi.de (M.R.); t416010@st.pu-toyama.ac.jp (S.K.); 2 German Centre for Infection Research (DZIF), Partner Site Hannover-Braunschweig, 38124 Braunschweig, Germany; 3 Zoological Institute, Technical University of Braunschweig, Spielmannstraße 7, 38106 Braunschweig, Germany; r.koester@tu-braunschweig.de; 4 Toyama Prefectural University, 5180 Kurokawa, Imizu-shi, Toyama 939-0398, Japan

**Keywords:** natural products, fungal metabolites, neurotrophic activity, fermentation, Basidiomycota

## Abstract

Basidiomycetes of the genus *Hericium* are among the most praised medicinal and edible mushrooms, which are known to produce secondary metabolites with the potential to treat neurodegenerative diseases. This activity has been attributed to the discovery of various terpenoids that can stimulate the production of nerve growth factor (*NGF*) or (as established more recently) brain-derived neurotrophic factor (*BDNF*) in cell-based bioassays. The present study reports on the metabolite profiles of a Lion’s Mane mushroom (*Hericium erinaceus*) strain and a strain of the rare species, *Hericium flagellum* (synonym *H. alpestre*). While we observed highly similar metabolite profiles between the two strains that were examined, we isolated two previously undescribed metabolites, given the trivial names erinacines Z1 and Z2. Their chemical structures were elucidated by means of nuclear magnetic resonance (NMR) spectroscopy and high resolution mass spectrometry. Along with six further, previously identified cyathane diterpenes, the novel erinacines were tested for neurotrophin inducing effects. We found that erinacines act on *BDNF*, which is a neurotrophic factor that has been reported recently by us to be induced by the corallocins, but as well on *NGF* expression, which is consistent with the literature.

## 1. Introduction

Neurotrophins such as nerve growth factor (*NGF*) and brain-derived neurotrophic factor (*BDNF*) are important for the survival, maintenance, and regeneration of specific neuronal populations in the adult brain. Therefore they can be used as a remedy in the fight against neurodegenerative diseases such as Parkinson’s, Alzheimer’s and Huntington’s diseases, which are accompanied by decreased neurotrophic factor expression [1]. Chemical constituents from *Hericium erinaceus* promote neuronal survival and potentiate neurite outgrowth via the TrkA/Erk1/2 pathway. Since neurotrophins are not able to pass the blood brain barrier (BBB) due to their high molecular weights, *NGF*-inducing small molecules and *NGF* mimics became the focus of research activities [2,3]. In the domain of natural product research, several *NGF*-inducing constituents that are derived from different sources have been reported [4,5], and among the first were secondary metabolites of *H. erinaceus*. This edible mushroom has a long history of usage in traditional Chinese medicine and is used in various food supplements and nutraceuticals [6,7]. From the basidiomes and corresponding mycelial cultures, various terpenoids were isolated that possessed immunomodulatory, anti-inflammatory, anti-proliferative, antimicrobial, and neuritogenic activities [7,8,9,10]. Interestingly, both the erinacine type cyathane diterpenoids that were produced in mycelial cultures and the hericenone type meroterpenoids that are located in the basidiomes of *H. erinaceus*, have been found to stimulate *NGF* synthesis [7]. We have recently discovered new compounds of the latter type from basidiomes of the related species *Hericium coralloides*, which were the first members of this compound family that modulated both, *NGF* and *BDNF* production. This prompted us to extend our studies to the metabolites from cultures of other *Hericium* species. The present study is dedicated to a comparison of the secondary metabolite profiles of two strains from this genus. Aside from a strain of *H. erinaceus* that was derived from a specimen that was collected in Southwest Germany, we used another strain of the rare species, *H. flagellum* (synonym: *H. alpestre*) that was deposited many years ago in a public culture collection. The latter species is associated with conifers, in particular *Abies*, and has to our knowledge never been studied for secondary metabolites. The terpenoids of both strains were isolated to purity and subsequently examined in our bioassays, in order to evaluate their neurotrophic activities.

## 2. Results and Discussion

### 2.1. Secondary Metabolites of Hericium erinaceus and Hericium flagellum

**Figure 1:**
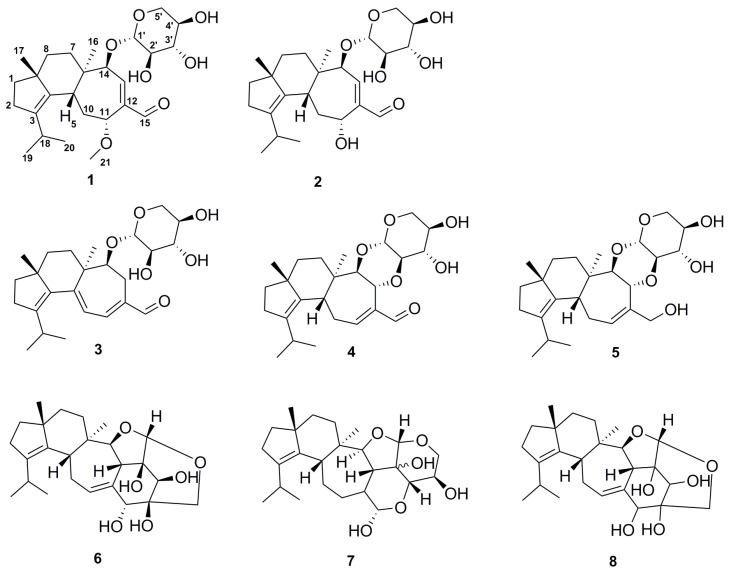
Chemical structures of the isolated cyathane diterpenoids (**1**–**8**).

Mycelial cultures of *H. erinaceus* and *H. flagellum* were examined for their secondary metabolites profiles and the results of this study are presented in Table 1. Two new erinacine derivatives (**1**) and (**2**) and six known compounds, erinacine A (**3**) [6], erinacine B (**4**) [6], erinacine C (**5**) [6], erinacine E (**6**) [11], CJ14.258 (**7**), and erinacine F (**8**) [11] were structurally characterized (Figure 1). Dominant compounds in the extracts of both the strains were cyathane diterpenoids (Table 1) with almost no difference in the variety of diterpenoids isolated. Although observed from *H. flagellum* only, **7** and **8** were reported to be produced by other species from the genus *Hericium* [11]. Further work involving additional strains and culture media will show whether there are specific metabolies that are produced by certain species of the genus; however, the current study confirmed the apparent specificity of cyathane diterpenes as prevailing metabolites of *Hericium* species.

### 2.2. Structure Elucidation of Compounds ***1**–**8***

The chemical structures of the new compounds were elucidated by Electrospray ionization-mass spectrometry (ESI-MS) and interpretation of two-dimensional (2D) NMR data. The known compounds were identified by comparing their spectroscopic data with those reported in the literature.

Compound (**1**) was obtained as colorless oil and exhibited a major peak in the High resolution-Electrospray ionization-mass spectrometry (HR-ESIMS) spectrum at *m*/*z* = 465.2853 ([M + H]^+^, calcd. for C_26_H_41_O_7_ [M + H]^+^; *m*/*z* = 465.2847), consistent with the molecular formula C_26_H_40_O_7_ indicating 7 degrees of unsaturation. The ^1^H NMR spectrum of (**1**) (Table 2) displayed four methyl signals at *δ*H 0.93 (3H, s), 0.99 (3H, s), 0.97 (6H, d, *J* = 6.8 Hz) and 3.26 (3H, s), which is characteristic of a methoxy group and twenty proton signals in the *δ*H 1.32–4.65 ppm range. In addition the ^1^H spectrum revealed one olefinic proton at *δ*H 6.94 (1H, dd, *J* = 5.6, 1.2 Hz) and a singlet at *δ*H 9.54 (1H, s) characteristic of an aldehyde. The ^13^C NMR and heteronuclear single quantum coherence- distortionless enhancement by polarization transfer (HSQC-DEPT) spectra implied the presence of five non-proton-bearing carbons, including three olefinic (*δ*C 139.9, 136.6, 138.6) and two aliphatic carbons (*δ*C 40.6, 49.2). Furthermore, five methylene groups with corresponding carbons between *δ*C 28.4–38.5 ppm, a further oxygenated methylene group at *δ*C 65.1, viscinal to two aliphatic methines between *δ*C 27.0 and *δ*C 40.4, and six methines at *δ*C 69.6–105.3 ppm were observed. A carbonyl carbon at *δ*C 192.9 with the corresponding proton signal at *δ*H 9.54 suggested the presence of an aldehyde moiety. ^1^H-^1^H-COSY (COrrelated SpectroscopY) and heteronuclear multiple bond correlation (HMBC) correlations clearly indicated a presence of a [5-6-7] tricyclic carbon skeleton with an isopropyl group connected to the olefinic carbon at *δ*C 139.9 (C-3) and an allylic aldehyde located at the seven-membered ring (C-13, C-12, C-15) in **1**.

**Figure 2:**
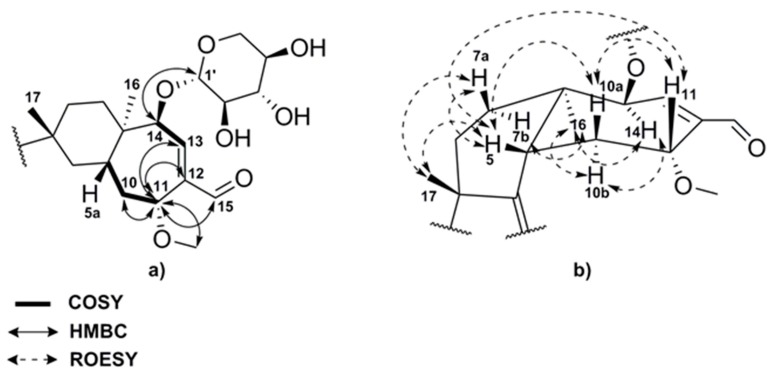
(**a**) Important ^1^H-^1^H-COSY and ^1^H-^13^C-HMBC correlations of erinacine Z1 **1**; (**b**) Relevant ROESY correlations of erinacine Z1 **1**.

Additionally, COSY correlations between the methylene protons at C-10 and the proton at *δ*H 4.63 as well as ^2^*J*-HMBC correlations of this proton to methylene carbon C-10 and olefinic carbon C-12 and ^3^*J*-HMBC correlations to the methoxy carbon C-21, the olefinic carbon C-13 and the carbonyl carbon C-15 pointed to a methoxy group next to the allylic aldehyde at C-11 (Figure 2a). Further studies of the 2D NMR data (HSQC, COSY, HMBC, and rotating-frame nuclear Overhauser effect correlation spectroscopy; ROESY) revealed a β-d-xylopyranose, which is commonly attached to cyathane diterpenoids via carbons C-13 and/or C-14. The downfield shift of the proton and carbon signals of the C-14 suggested a position adjacent to an oxygen atom and the HMBC correlation to methine (C-1′) of the xyloside led to the structure of compound **1**.

The comparison of the NMR signals of **1** with literature values of structurally similar cyathane xylosides such as erinacine P [12] confirmed its structure. The relative stereochemical structure of **1** was determined by ROESY and NOESY experiments, which exhibited correlations of proton H-5, methyl group CH_3_-17, methylene protons H-7a and H-10a as well as methine proton H-11. On the other hand correlations between methyl group CH_3_-16, methine proton H-14 and methylene protons H-7b and H-10b could be observed (Figure 2b).

Compound (**2**) was also isolated as colorless oil. The [M + H]^+^ peak at *m*/*z* =451.2689 ([M + H]^+^, calcd. for C_25_H_39_O_7_ [M + H]^+^, *m*/*z* = 451.2690) in the HR-ESI-MS spectrum provided the molecular formula C_25_H_38_O_7_ with 7 degrees of unsaturation. The ^1^H NMR data of **2** revealed four methyl groups at *δ*_H_ 0.82 (3H, s), *δ*_H_ 0.97 (6H, m), and *δ*_H_ 1.14 (3H, s), twenty protons in the *δ*_H_ 1.16–4.78 ppm range, an olefinic proton at *δ*_H_ 7.01 (1H, dd, *J* = 7.3, 0.9 Hz), and a singlet at *δ*_H_ 9.43 (1H, s), similar to compound **1**. Also, the ^13^C shifts and correlations of the HSQC-DEPT spectrum showed high similarity to **1** and suggested that compound **2** was a derivative of the cyathane diterpenoid **1**. The major difference between the two compounds spectra was the missing methoxy group at C-11 in **2**. The analysis of the COSY and HMBC data revealed the same proton-proton and proton-carbon long range correlations of methine H-11 at *δ*_H_ 4.78 (1H, br d, *J* = 5.2 Hz) and *δ*_C_ 62.1 as in compound **1**, but the high field shift of the carbon signal and the compound mass of **2** were consistent with the presence of a hydroxyl group at C-11 instead of a methoxy moiety. Similar to compound **1**, the ROESY spectrum displayed correlations between proton H-5, methyl group CH_3_-17, methylene proton H-10a and methine proton H-11, indicating that all of them have the same stereochemical orientation. Additionally, ROE correlations between the methyl group CH_3_-16 and the methine proton H-14 could be observed, which finally led to the 11-hydroxy derivative **2**.

### 2.3. NGF-Induced Neurite Outgrowth in PC12 Cells

**Figure 3:**
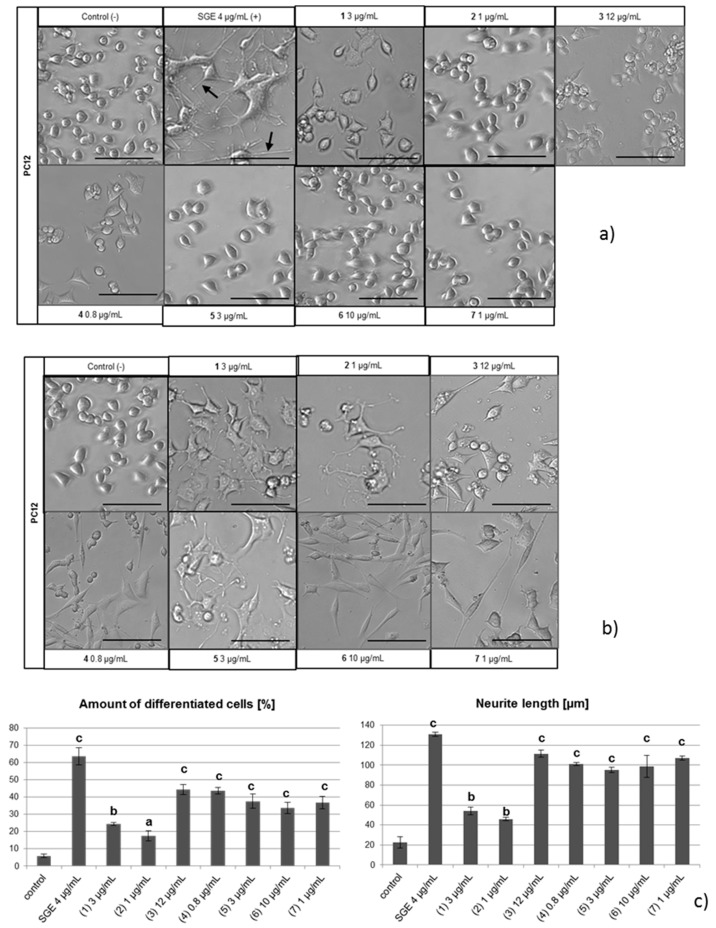
Morphological differentiation of PC12 cells incubated with erinacines Z1, Z2, A, B, C, E, and CJ14.258 (**1**–**7**) (**a**) or conditioned medium produced by 1321 N1 cells; (**b**) (−): negative control, no additive; (−) Ethanol: negative control with ethanol; (−) 1321N1 medium: negative control with 1321N1 medium; (+) SGE: positive control with salivary gland extract (contains *NGF*). Differentiated cells are marked with an arrow; (**c**) a quantification of analyses with the amount of differentiated cells [%] or neurite length [µm]. Scale bar 100 µm (±SEM; a, *p* < 0.01; b, *p* < 0.001; c, *p* < 0.0001).

All of the isolated metabolites were evaluated for their neurotrophin inducing effects. Immortalized PC12 cells derived from rat adrenal medullary tumor (pheochromocytoma) cells and that are widely employed as an in vitro model for neuronal differentiation and neurite outgrowth studies were used. PC12 cells require *NGF* to differentiate, upon which they stop proliferating, extend neurites, and become electrically excitable [13]. A direct neurotrophic effect of the test compounds was analyzed by incubating PC12 cells in the highest non-toxic concentration of each compound in serum-free medium. Murine salivary gland extract, which is known to contain *NGF*, was used as a positive control causing PC12 cell differentiation. In this experimental setup, no signs of neurite outgrowth from PC12 cells were observed (Figure 3a), indicating that none of the tested diterpenoids exerted a direct neurotrophic effect.

Since PC12 cells do not produce *NGF* themselves [13], we used human 1321N1 astrocytoma cells to test for the induction of cyathane-induced *NGF* secretion. As a negative control, ethanol was used according to the final concentration of the dissolved compounds. Subsequently, the conditioned astrocyte media were used to incubate PC12 cells. All tested compounds displayed PC12 differentiation activity triggering neurite outgrowth (Figure 3b) with respect to both the number of differentiating PC12 cells as well as the length of induced neurite outgrowth (Figure 3c). These findings suggest that the metabolites isolated from cultures of *Hericium erinaceus* and *flagellum* possess neurotrophin-inducing properties in astrocytic cells with compounds **3**, **4**, **5**, **6**, and **7** being most potent. The new metabolites **1** and **2** exhibited relatively weak effects on PC12 cell differentiation.

### 2.4. Semi-Quantitative Reverse Transcriptase PCR to Quantify the Neurotrophin mRNA Level

**Figure 4:**
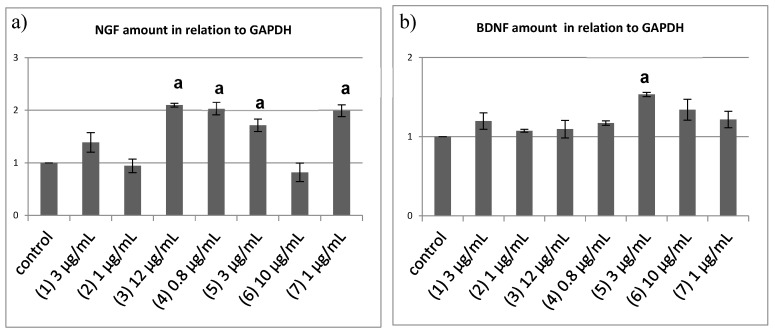
Semiquantitative RT-PCR analysis for mRNA of (**a**) nerve growth factor (*NGF*) and (**b**) brain-derived neurotrophic factor (*BDNF*); (**a**) A significant increase of *NGF* was observed for several cyathane derivatives tested. Compounds **3**, **4,** and **7** increased *NGF* mRNA by two times, compounds **5** increase *NGF* by 1.6 times, whereas **2** and **6** failed to induce *NGF* mRNA upregulation; (**b**) Only 1321N1 incubated with **5** show a significantly higher *BDNF* mRNA amount when compared to the ethanol control (±SEM; a, *p* < 0.05).

To directly confirm induction of neurotrophin expression in 1321N1 cells by tested compounds 1321N1 cells were incubated with these compounds for 6 h, followed by RNA isolation, cDNA synthesis, and semi-quantitative RT-PCR analysis. Ubiquitously expressed glyceraldehyde 3-phosphate dehydrogenase (*GAPDH*) was used as a reference to determine relative amounts of *NGF* (Figure 4a) and *BDNF* induction (Figure 4b), respectively. Supporting the results of the PC12 differentiation assays, compounds **3**, **4**, **5**, **7**, and but also the new derivative **1** showed an increase of *NGF* mRNA between 1.4 and 2 folds. Out of those, compounds **3**, **4**, **5** and **7** induced higher levels of *NGF* expression than compound **1**, in agreement with the results of the PC12 differentiation assays. Likewise, compound **2** treatment did not result in the induction of *NGF* expression consistent with its poor performance in the PC12 differentiation assay. Intriguingly **6** did not result in the induction of elevated *NGF* expression in contrast to its strong neurite extension activity in the PC12 assay. Either **6** induces *NGF* expression in 1321N1 cells with a lag phase or currently unknown factors with PC12 differentiation-inducing activity are released into the medium by **6** treated astrocytic cells.

Another prominent neurotrophic factor is *BDNF*; but because PC12 cells do not express its high-affinity receptor tropomyosin receptor kinase B (TrkB) [14,15], *BDNF* is not able to trigger neurite outgrowth in PC12 cells. Semi-quantitative RT-PCR revealed that of all applied compounds only **5** harbors *BDNF*-inducing properties at moderate levels, similar to the recently characterized corallocins [6]. In addition to that, erinacines contain different neurotrophin-inducing signatures, with some being able to induce a cocktail of neurotropohin-secretion from astrocytes.

Compounds **3**, **4**, **5**, **7**, and the novel derivative **1** were found to act on the 1321N1 cells by increasing the transcription of either *BDNF* or *NGF*. Among all of the compounds that were tested, **6** and **2** failed to induce neurotrophin production.

## 3. Materials and Methods

### 3.1. General Experimental Procedures

One-dimensional (1D) and 2D NMR spectra were recorded on an Avance III 700 spectrometer (Bruker, Bremen, Germany); with a 5 mm TXI cryoprobe (^1^H 700 MHz, ^13^C 175 MHz) or an Avance III 500 (^1^H 500 MHz, ^13^C 125 MHz) spectrometer (Bruker); optical rotations were measured on a Perkin-Elmer 241 polarimeter, IR spectra were measured with a Nicolet Spectrum 100 FTIR spectrometer (Perkin-Elmer, Waltham, MA, USA). All of the HPLC analyses were performed on a 1200 Series (Agilent, Santa Clara, CA, USA), equipped with degasser, Bin Pump SL (Agilent technologies 1260 Infinity), autosampler and DAD/electron light scattering detector Corona Ultra RS. C18 Acquity UPLC BEH column (2.1 × 50 mm, 1.7 μm; Waters, Eschborn, Germany) was used. Solvent A: H_2_O + 0.1% formic acid, solvent B: acetonitrile + 0.1% formic acid with the following gradient: 0–0.5 min 5% B, 0.5–20.0 min 100% B, 20–30 min 100% B; injection volume was 2 µL, flow rate 600 µL/min; UV/Vis detection 200–600 nm.

LC-ESI-MS spectra were recorded on an ion trap mass spectrometer (scan range 100–2000 *m*/*z*, capillary voltage 4000 V, dry temperature 250 °C) (amaZon speed, Bruker), and HR-ESI-MS spectra on a time-of-flight (TOF) mass spectrometer (scan range 250–25000 *m*/*z*, capillary voltage 4500 V, dry temperature 200 °C) (MaXis, Bruker).

The chemicals and solvents were obtained from AppliChem GmbH (Darmstadt, Germany), Avantor Performance Materials (Deventor, The Netherlands), Carl Roth GmbH & Co. KG (Karlsruhe, Germany), and Merck KGaA (Darmstadt, Germany) in analytical and HPLC grade.

### 3.2. Fungal Material and Fermentation

*Hericium erinaceus* strain STMA 06157B was isolated from the basidiome of a specimens that were collected by Benno Stadler in Germany, Rhenania-Palatinate Province, near Eppenbrunn on *Fagus sylvatica* in September of 2006. The culture and the corresponding specimen are deposited at the herbarium and culture collection of the DSMZ, Braunschweig, Germany. *Hericium flagellum* (deposited as “*H. alpestre*” strain CBS 103681 had been originally isolated by N. Hallenberg from a specimens collected from *Abies* in Romania and was purchased from Westerdijk Fungal Biodiversity Centre (Utrecht, The Netherlands).

A seed culture of *H. erinaceus* was prepared as follows: five mycelial plugs (0.5 × 0.5 cm^2^) were cut from well grown yeast-malt-glucose agar (YMG) plates and were inoculated in 2 × 100 mL ZM/2 medium [16]. Flasks were incubated on a rotary shaker for 168 h at 24 °C and 160 rpm. 4 × 40 mL of the first inoculum was added in 4 × 1000 mL sterile Erlenmeyer flasks with 400 mL working volume of ZM/2 medium and incubated at the same conditions. A 10 L batch bioreactor, containing ZM/2 medium, equipped with temperature control, pH controller, and agitation, was inoculated with 10% of the seed culture, fermented for 72 h at 24 °C and 150 rpm. Finally it served as a seed culture for a 70 L bioreactor (10% seed culture added, fermented for 192 h at 24 °C and 150 rpm).

*Hericium flagellum* was fermented in two different media, ZM/2 and SP [d(+) glucose monohydrate 30 g/L, soy peptone 6 g/L, KH_2_PO_4_ 2.5 g/L, MgSO_4_ 0.5 g/L, yeast extract 1.5 g/L, CaCl_2_·2H_2_O 73.5 mg/L, pH 6.0; after autoclaving 1 mL/L trace element solution sterile filtrated-FeCl_3_·6H_2_O 80 mg/L, MnSO_4_·H_2_O 30 mg/L, ZnSO_4_·H_2_O 90 mg/L, EDTA 400 mg/L]. Fifteen 500 mL Erlenmeyer flasks containing 200 mL media were used for each media and later incubated on a rotary shaker for 97 days (ZM/2 medium) and 60 days (SP medium), respectively, at 24 °C and 160 rpm.

### 3.3. Extraction and Isolation

The biomass (1240 g) of *H. erinaceus* was extracted with 6L of acetone according to the protocol of Chepkirui et al. [17] to yield 12.5 g of the crude extract, whereas the culture broth was incubated with 1% (*v*/*v*) of Amberlite XAD™-16N adsorber resin (Sigma-Aldrich, Deisenhofen, Germany) under stirring for 1 h. The resin was thereafter removed by filtration and subsequently extracted, according to the aforementioned extraction protocol [17], to give 6 g of crude extract.

The biomass and supernatant of *H. flagellum* were separated by filtration and extracted as described by Chepkirui et al. [17]. From the fermentation carried out in ZM/2 medium 454 mg and 65 mg of crude extracts were obtained from supernatant and biomass, respectively.

The crude mycelial extract obtained from *H. erinaceus* was subjected to Flash chromatography on a Reveleris X2 system (Grace, Columbia, MD, USA) using a normal phase Reveralis 80 g Silica Cartridge (Grace) as stationary phase, while the mobile phase was composed of n-heptane (solvent A) and methyl tert-butyl ether (TBME, solvent B). A linear gradient from 0% to 100% B in 40 min and flow rate of 60 mL/min were applied to elute compound **4** (242 mg) at *t_R_* =15 min and 22% of solvent B. UV detection at 220, 254 and 310 nm, together with electron light scattering detection (ELSD) were used.

The supernatant crude extract of *H. erinaceus* was fractionated by the same Flash chromatography system, as described above. A reversed phase Reveralis 80 g C18 Flash Cartridge (Grace, Columbia, MD, USA) was used as a stationary phase and a mixture of deionized water (solvent A) and acetonitrile (ACN, solvent B) were used as mobile phase. The separation was carried out according to the following gradient: 35% B isocratic for 5 min, followed by a linear increase to 80% over 50 min, afterwards increasement to 100% of B in 5 min, and thereafter isocratic conditions at 100% for 5 min. Flow rate of 60 mL/min was applied; UV detection at 220, 254 and 310 nm and ELSD were used. Eight fractions (S1–S8) were collected and combined to the observed peaks.

Fraction S6 (312 mg), obtained at *t_R_* =23 min and 52% of B was further purified on preparative HPLC using Gilson GX270 Series HPLC system (Gilson Inc., Middleton, WI, USA) and a Nucleodur C18 column (10 µm; 150 × 40 mm, Macherey Nagel, Düren, Germany) as stationary phase. The mobile phase consisted of solvent A: H_2_O + 0.1% triflouroacetic acid and solvent B: ACN + 0.1% triflouroacetic acid. A gradient was run from 52% B to 75% B in 50 min, with a flow rate 30 mL/min, to give compound **5** (22 mg) at *t_R_* =30 min and 62% of B whereas the fraction (obtained at *t_R_* =23 min and 61% B) was repeatedly purified using the same HPLC protocol and a Kromasil C18 column (7 µm; 250 × 21.5 mm, MZ Analysentechnik, Mainz, Germany) as stationary phase to afford the compound **3** (2.24 mg) at *t*_R_ = 22 min and 69% of B.

Furthermore, fraction S7 (965 mg) obtained at *t_R_* = 29 min and 57% of B was subjected to the reversed phase flash chromatography system that is described above, and the separation was carried out according to the same protocol that was used for the fractionation of the crude extract of the supernatant of *H. erinaceus*. The fraction eluted at *t_R_* = 20 min and 67% of B (627 mg) and was further purified on preparative HPLC using the same conditions as stated above for fraction S6. Compound **1** (6.7 mg) was obtained at *t_R_* = 17 min and 58% of B, whereas the fraction eluted at *t_R_* = 35 min and 68% of B was further purified with the same mobile phase composition and the gradient from 54% of B to 70% in 40 min, flow rate of 15 mL/min and with a Kromasil C18 column (7 µm; 250 × 21.5 mm, MZ Analysentechnik) as stationary phase to give **6** (1.8 mg) at *t_R_* = 31 min and 74% of B.

The supernatant and mycelial extracts of *H. flagellum* obtained from the fermentation in ZM/2 media were combined and pre-purified using solid phase extraction. A Strata™-X 33 µm Polymeric Reversed Phase cartridge (Phenomenex, Torrance, CA, USA) was used as a stationary phase, while methanol (MeOH) was used as a mobile phase. Thereafter, the same preparative HPLC protocol described for S6 was employed to afford **2** (2.7 mg) at *t_R_* = 15 min and 55% of B.

The crude extracts that were obtained from *H. flagellum* fermented in SP media were pre-purifed as stated above and fractionated using the same HPLC system with a gradient from 20% B to 80% B in 60 min and flow rate of 14 mL/min. As stationary phase, a Kromasil C18 column (7 µm; 250 × 21.5 mm) was used. Compounds **7** (1 mg) and **8** (0.3 mg) eluted at *t_R_* = 46 min and *t_R_* = 38 min, respectively.

### 3.4. Differentiation Assay

1321N1 astrocyte cells were grown in Gibco™ DMEM (Fischer Scientific, Hampton, NH, USA) medium containing 10% fetal bovine serum (FCS), and PC12 cells were cultivated in Gibco™ RPMI 1640 (Fischer Scientific, Hampton, NH, USA) medium containing 10% horse serum (HS) and 5% fetal bovine serum (FCS). In addition, both media were supplemented with penicillin (0.15 mM), streptomycin (86 μM), and glutamine (2 mM). About 48 h before the compounds were added, 1321N1 cells were seeded on 6-well plates with a density of 1 × 10^5^ cells/well and incubated at 37 °C with 5% CO_2_. 24 h before adding the compounds, serum free DMEM supplemented with penicillin (0.15 mM), streptomycin (86 μM), and glutamine (2 mM) was added. After adding the test compounds in different concentrations the cells were incubated for another 48 h to produce conditioned media. As control, 0.5% ethanol in water was used.

PC12 cells were seeded at a density of 4 × 10^4^ cells/well on a 24-well plate coated with 0.005% collagen (Roche, Basel, Switzerland) and incubated at 37 °C with 5% CO_2_. After 48 h, either the compounds or the conditioned media were added. PC12 cells were analyzed by transmitted light microscopy for neurite outgrowth. Neurites longer than one cell diameter indicated differentiated cells. Three independent experiments were performed with approximately 100 cells analyzed each.

### 3.5. Reverse Transcriptase PCR

For semiquatitative reverse transcriptase assays 1321N1 astrocytes were seeded and incubated as described above. After incubation with the compounds for six hours, total RNA was isolated from 1321N1 cells using RNAPure™, peqGOLD (PEQLAB Biotechnologie, Erlangen, Germany). First-strand cDNA synthesis was performed from 1 μg of the total RNA using AMV reverse transcriptase (Promega, Madison, WI, USA) and oligo (dT) primer (Promega, Madison, WI, USA). The following PCR primers were used for amplification which resulted in amplified PCR-fragments of the following sizes: *GAPDH* (sense: 5′-TCCACCACCCTGTTGCTGTA-3′; antisense: 5′-CCACAGTCCATGCCATCAC-3′; 451 bp), *NGF* (sense: 5′-CCAAGGGAGCAGTTTCTATCCTGG-3′; antisense: 5′-GCAGTTGTCAAGGGAATGCTGAAGTT-3′; 189 bp), *BDNF* (sense: 5′-TAACGGCGGCAGACAAAAAGA-3′; anti-sense: 5′-GAAGTATTGCTTCAGTTGGCCT-3′; 101 bp). PCR was performed in a 25 μL volume containing cDNA template (2 μL), dNTP (10 mM, 0.5 μL), primers (100 μM; 0.1 μL), Go Taq buffer (5×, 5 μL), and Go Taq polymerase (5 U/μL; 0.2 μL, Promega, Madison, WI, USA). The amplification programs were started with a step of 94 °C for 2 min and finished by a 72 °C step for 5 min, while following cycles of amplification were used: GAPDH, 20 cycles: 94 °C, 30 s, 60 °C, 30 s, 72 °C, 45 s; *NGF*, 30 cycles: 94 °C, 30 s, 61 °C, 30 s, 72 °C, 30 s; *BDNF*, 30 cycles: 94 °C, 30 s, 58 °C, 30 s, 72 °C, 30 s. *GAPDH* was used as loading control. The mRNA levels of *GAPDH, NGF*, and *BDNF* were analyzed on a 1% or 2% agarose gel. For comparing the amount of mRNA, FIJI was used [18].

## 4. Conclusions

In the course of our investigation of secondary metabolites from *Hericium* spp., two new cyathane diterpenoid derivatives together with several known metabolites were isolated from the submerged cultivations of *H. erinaceus* and *H. flagellum*. The latter species is very rare and has to the best of our knowledge never been studied for secondary metabolites. Seven metabolites from both strains were isolated to purity and were evaluated for their neurotrophin inducing effects. Although none of the tested compounds showed intrinsic neurothrophic activity, stimulating neurite outgrowth directly from cultured PC12 cells, compounds **3**, **4**, **7**, and the new derivative **1**, clearly enhanced neurotrophin production in astrocytic cells and can therefore be considered for an upcoming in vivo evaluation. Moreover, for the first time we observed a promoting effect of cyathane diterpenoid derivatives on *BDNF* expression. Since erinacines are able to stimulate the transcription of both investigated neurothrophins, we suggest an upstream target, which is common to upstream events of both, *NGF* and *BDNF* induction. *NGF* and *BNDF* modulatory activities have recently also been reported from an erinacine containing extract of *H. erinaceus* [19], and the authors have emphasized the potential of the fungal extracts for therapy.

## Figures and Tables

**Table 1:** Comparison of secondary metabolites isolated from the cultures of *H. erinaceus* and *H. flagellum*.

Compounds	*H. erinaceus*	*H. flagellum*
Erinacine Z1 (**1**)	+	−
Erinacine Z2 (**2**)	−	+
Erinacine A (**3**)	+	+
Erinacine B (**4**)	+	+
Erinacine C (**5**)	+	+
Erinacine E (**6**)	+	+
CJ14.258 (**7**)	−	+
Erinacine F (**8**)	−	+

**Table 2:** ^1^H and ^13^C NMR data of erinacine Z1 (**1**; 500 MHz and 125 MHz in CDCl_3_) and erinacine Z2 (**2**; 700 MHz and 175 MHz in acetone-*d*_6_). Corresponding spectra are deposited in the Supplementary Materials.

Position	(1)		(2)	
	*δ*_C_, Type	*δ*_H_, m (*J* in Hz)	*δ*_C_, Type	*δ*_H_, m (*J* in Hz)
1	38.5, CH_2_	1.46, m; 1.62, m	38.3, CH_2_	1.54, m
2	28.4, CH_2_	2.27, m	29.1, CH_2_	2.31, m
3	139.9, C		140.7, C	
4	136.6, C		139.8, C	
5	40.4, CH	1.93, m	36.1, CH	3.47, m
6	40.6, C		44.5, C	
7a	30.4, CH_2_	1.32, td (12.2, 6.6)	34.3, CH_2_	1.16, m
7b		1.62, m		2.41, td (13.8, 4.2)
8	37.0, CH_2_	1.46, m	37.1, CH_2_	1.44, ddd (12.5, 7.7, 3.0) 1.54, m
9	49.2, C		50.4, C	
10b	30.1, CH_2_	1.81, ddd (14.2, 11.9, 5.5)	35.0, CH_2_	2.07, m
10a		2.54, dd (13.7, 10.1)		2.14, m
11	72.4, CH	4.63, m	62.1, CH	4.78, br d (5.2)
12	138.6, C		148.2, C	
13	158.2, CH	6.94, dd (5.6, 1.2)	154.6, CH	7.01, dd (7.3, 0.9)
14	85.4, CH	4.61, d (5.6)	85.3, CH	3.96, d (7.3)
15	192.9, CH	9.54, s	194.2, CH	9.43, s
16	16.4, CH_3_	0.99, s	16.4, CH_3_	0.82, s
17	24.5, CH_3_	0.93, s	24.7, CH_3_	1.14, s
18	27.0, CH	2.85, m	27.7, CH	3.08, m
19	21.5, CH_3_	0.97, d (6.8)	22.0, CH_3_	0.97, m
20	21.8, CH_3_	0.97, d (6.8)	22.3, CH_3_	0.97, m
21	56.6, CH_3_	3.26, s	-	
1′	105.3, CH	4.34, d (7.0)	106.3, CH	4.56, d (5.6)
2′	73.5, CH	3.47, m	74.6, CH	3.51, m
3′	69,6, CH	3.74, td (8.8, 5.1)	70.3, CH	3.51, m
4′	75.7, CH	3.54, dd (8.8)	73.3, CH	3.41, dd (7.3, 5.8)
5′	65.1, CH_2_	3.29, m 3.99, dd (11.7, 5)	65.0, CH_2_	3.28, dd (11.8, 7.5) 3.88, dd (11.8, 3.3)

## Supplementary Materials

Supplementary materials can be found at http://www.mdpi.com/1422-0067/19/3/740/s1.

## Abbreviations

NGF	Nerve growth factor
BDNF	Brain derived neurothrophic factor
ESI-MS	Electrospray ionization-mass spectrometry
NMR	Nuclear Magnetic Resonance
HR-ESI-MS	High resolution-Electrospray ionization-mass spectrometry
GAPDH	Glyceraldehyde 3-phosphate dehydrogenase
ZM	Sugar medium
SP	Soy peptone
IR	Infrared
DAD	Diode array detector
LC	Liquid chromatography
EDTA	Ethylene-diamine-tetra-acetic acid
DMEM	Dulbecco’s modified eagle’s medium
RPMI	Roswell park memorial institute medium
