# Supplementary material for: Two New Cyathane Diterpenoids from Mycelial Cultures of the Medicinal Mushroom Hericium erinaceus and the Rare Species, Hericium flagellum
Source: Int J Mol Sci. 2018 Mar 6;19(3):740. doi: 10.3390/ijms19030740 (PMC5877601; doi:10.3390/ijms19030740)

Supplementary Information

Two New Cyathane Diterpenoids from Mycelial Cultures of the Medicinal Mushroom *Hericium erinaceus* and the Rare Species, *Hericium flagellum*

Zeljka Rupcic [1,2†], Monique Rascher[1,2,3†], Sae Kanaki[1,4], Reinhard W. Köster[3], Marc Stadler[1,2,*] and Kathrin Wittstein[1,2,*]

1 Department Microbial Drugs, Helmholtz Centre for Infection Research GmbH, Inhoffenstraße 7, 38124 Braunschweig, Germany; zeljka.rupcic@helmholtz-hzi.com, monique.rascher@helmholtz-hzi.de, kathrin.wittstein@helmholtz-hzi.de, marc.stadler@helmholtz-hzi.de
2 German Centre for Infection Research (DZIF), partner site Hannover-Braunschweig, 38124 Braunschweig, Germany; zeljka.rupcic@helmholtz-hzi.com, monique.rascher@helmholtz-hzi.de, kathrin.wittstein@helmholtz-hzi.de, marc.stadler@helmholtz-hzi.de
3 Zoological Institute, Technical University of Braunschweig, Spielmannstraße 7, 38106 Braunschweig, Germany; r.koester@tu-braunschweig.de
4 Toyama Prefectural University, 5180 Kurokawam Imizu-shi, Toyama 939-0398, Japan; t416010@st.pu-toyama.ac.jp
* Correspondence: kathrin.wittstein@helmholtz-hzi.de, Tel.: +49 0531 6181-1930 and marc.stadler@helmholtz-hzi.de, Tel.: +49 0531 6181-4240

Content:

NMR spectra of Erinacines Z1 and Z2 (Figures S1-13)

Figure S1: ^{1}H-NMR spectrum (500 MHz, $CDCl_3$) of Erinacine Z1 (1)

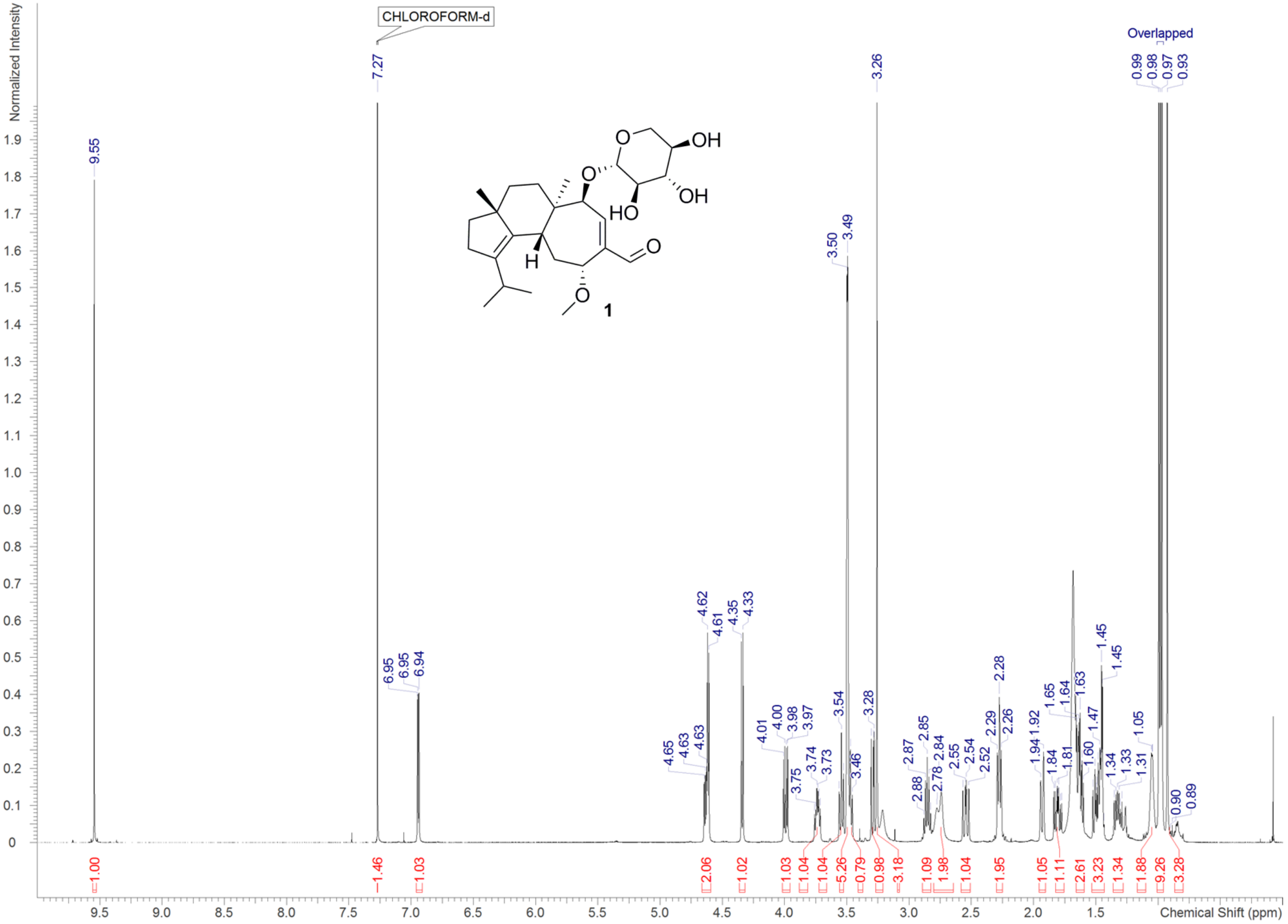

Figure S2: ¹³C-NMR spectrum (500 MHz, $CDCl_3$) of Erinacine Z1 (1)

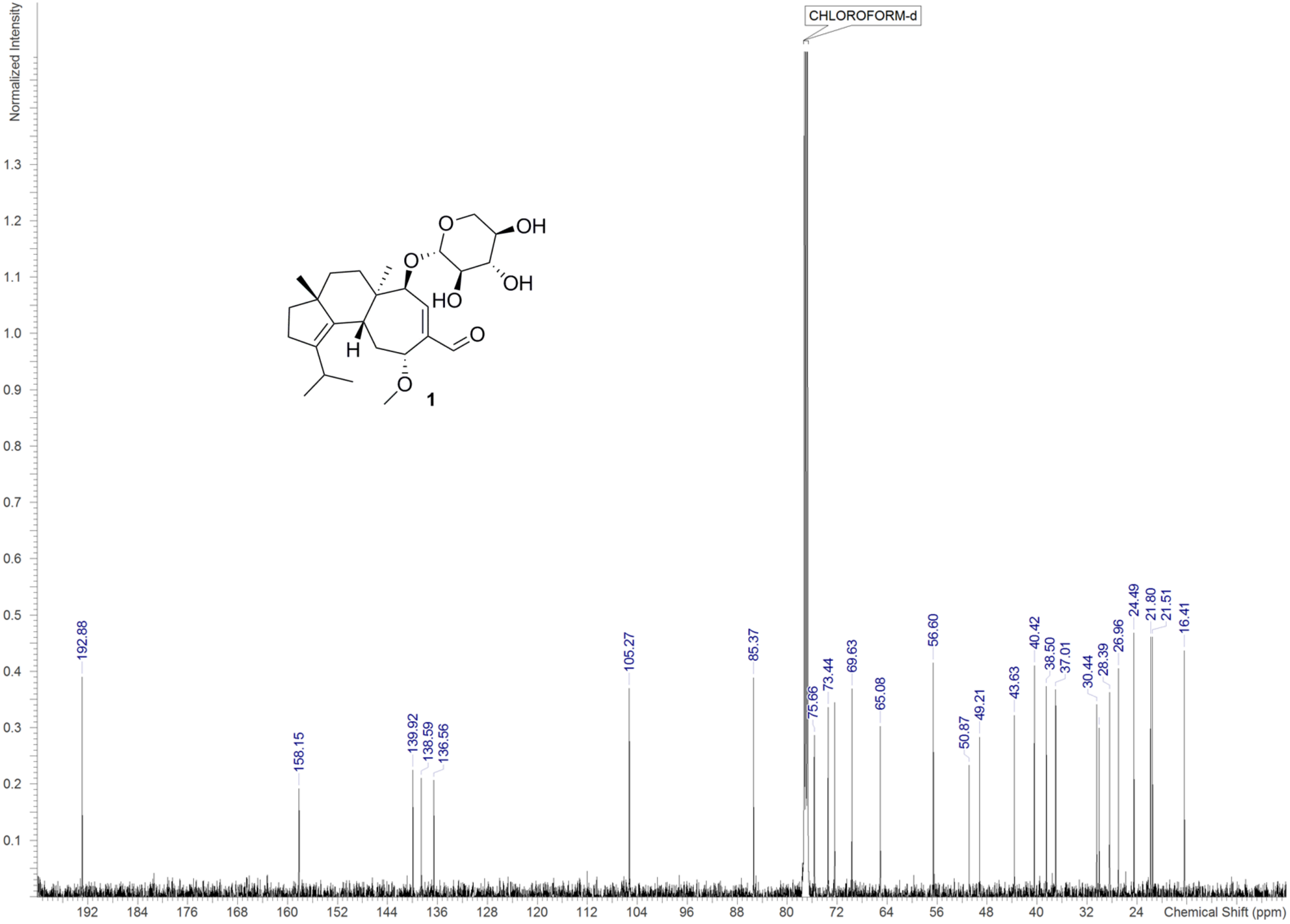

Figure S3: COSY spectrum (500 MHz, $CDCl_3$) of Erinacine Z1 (1)

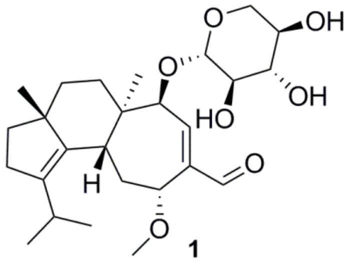

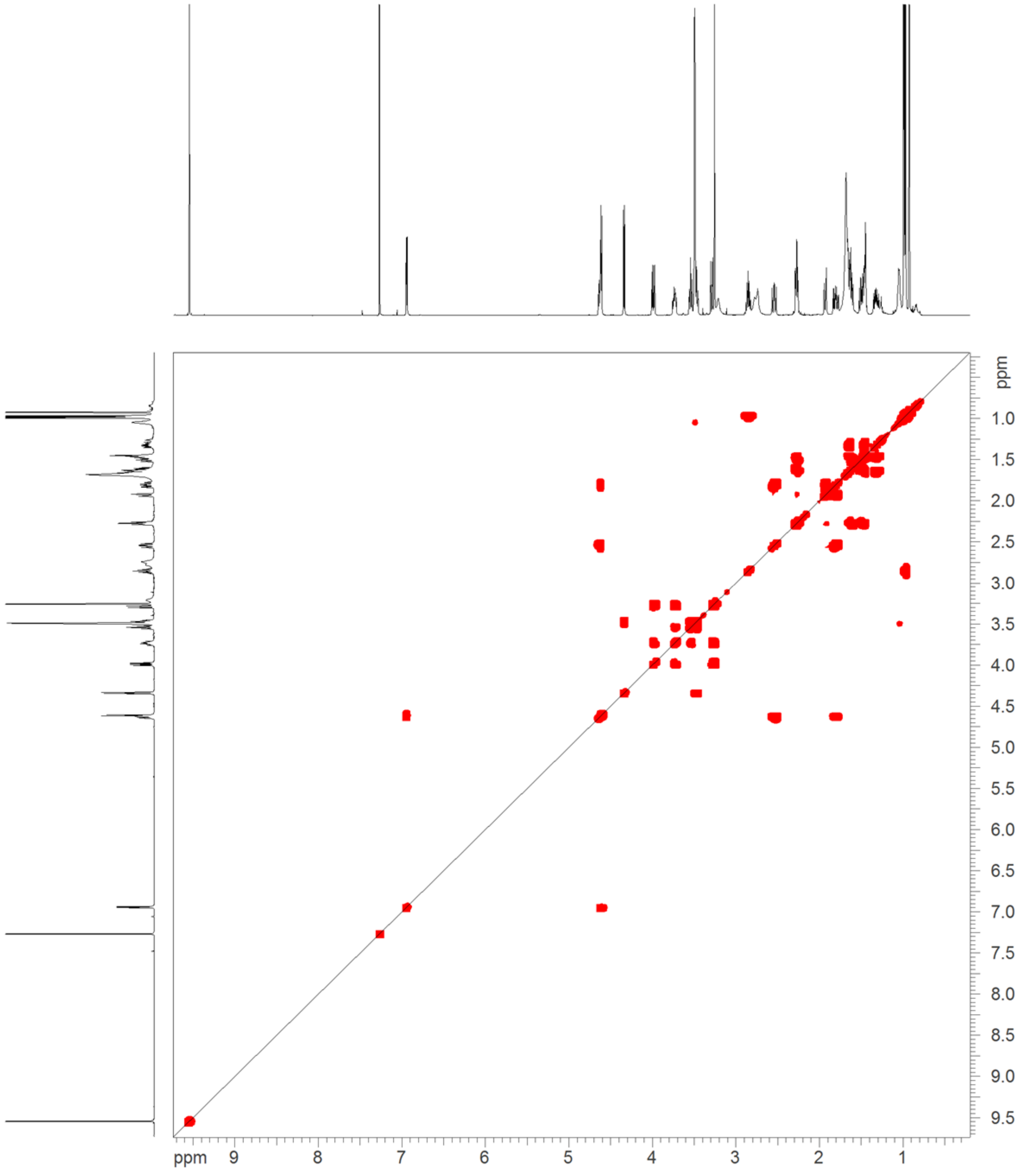

Figure S4: HSQC spectrum (500 MHz, CDCl$_3$) of Erinacine Z1 (1)

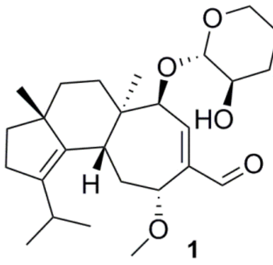

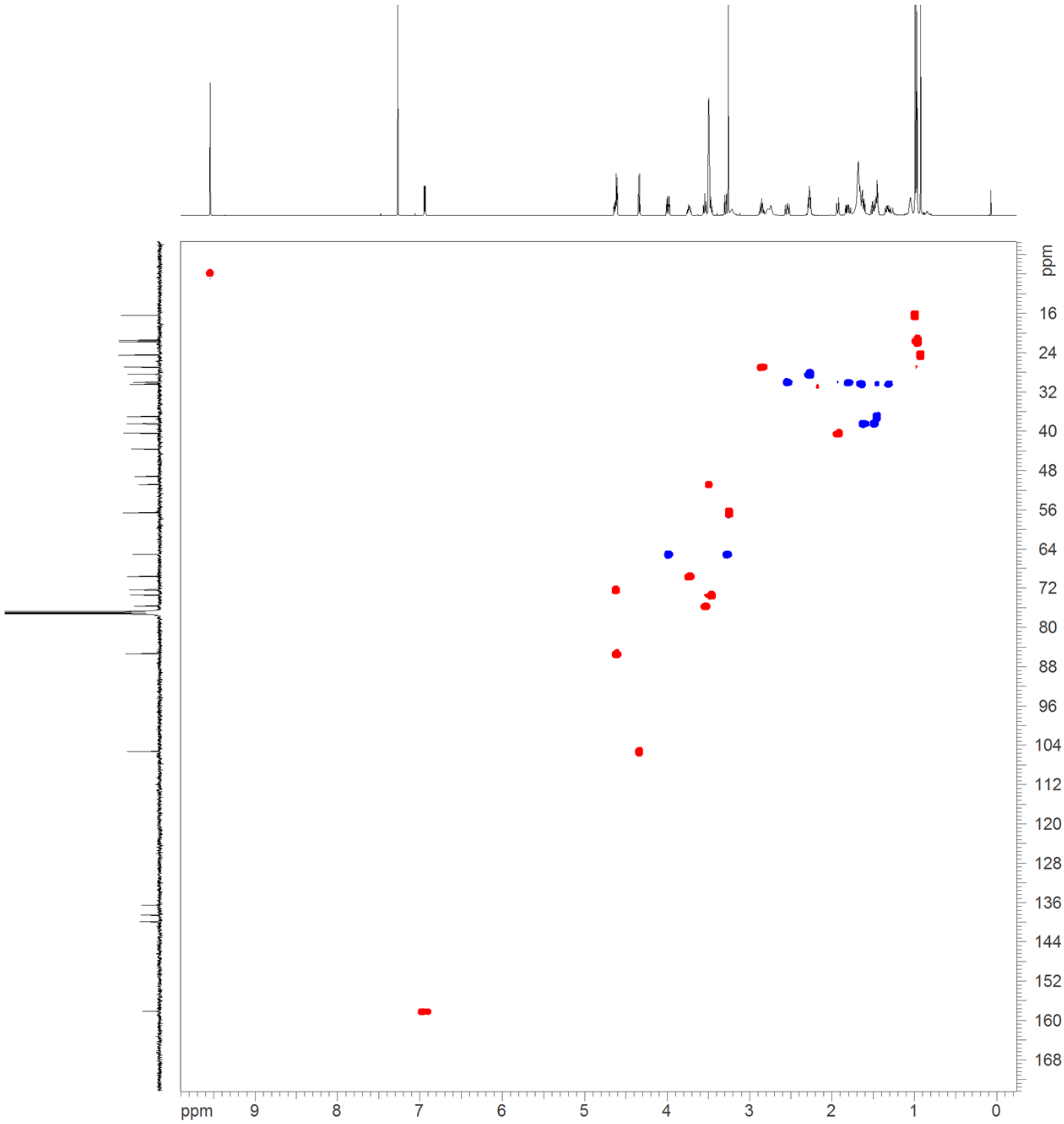

Figure S5: HMBC spectrum (500 MHz, $CDCl_3$) of Erinacine Z1 (1)

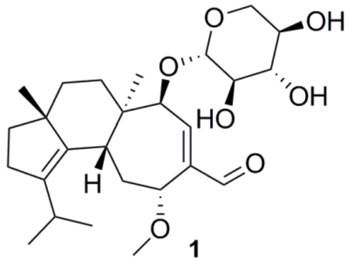

1

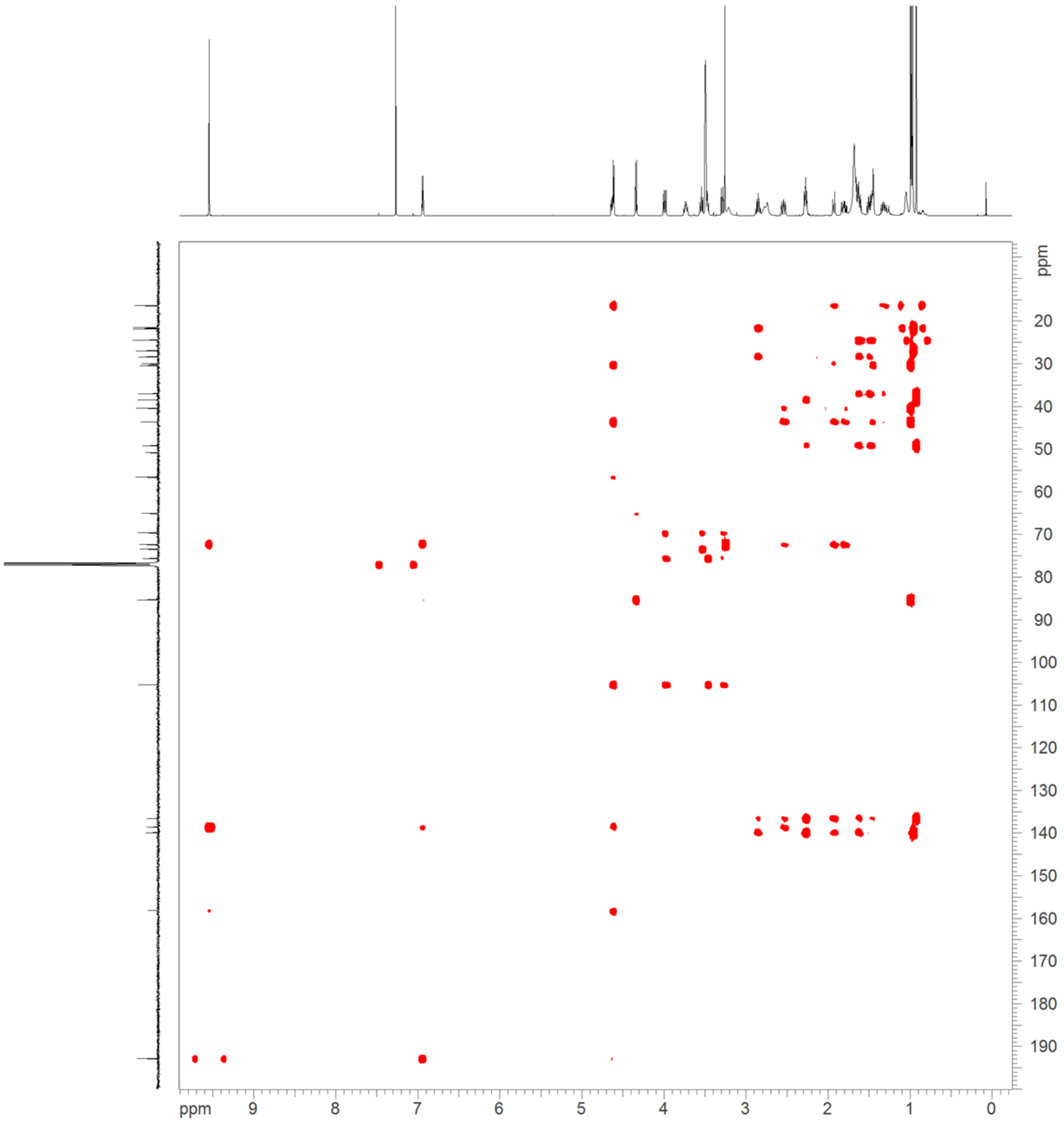

Figure S6: ROESY spectrum (500 MHz, $CDCl_3$) of Erinacine Z1 (1)

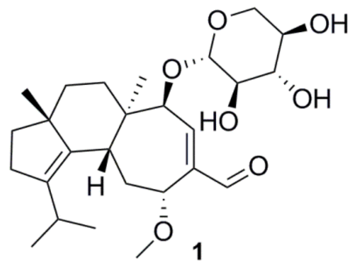

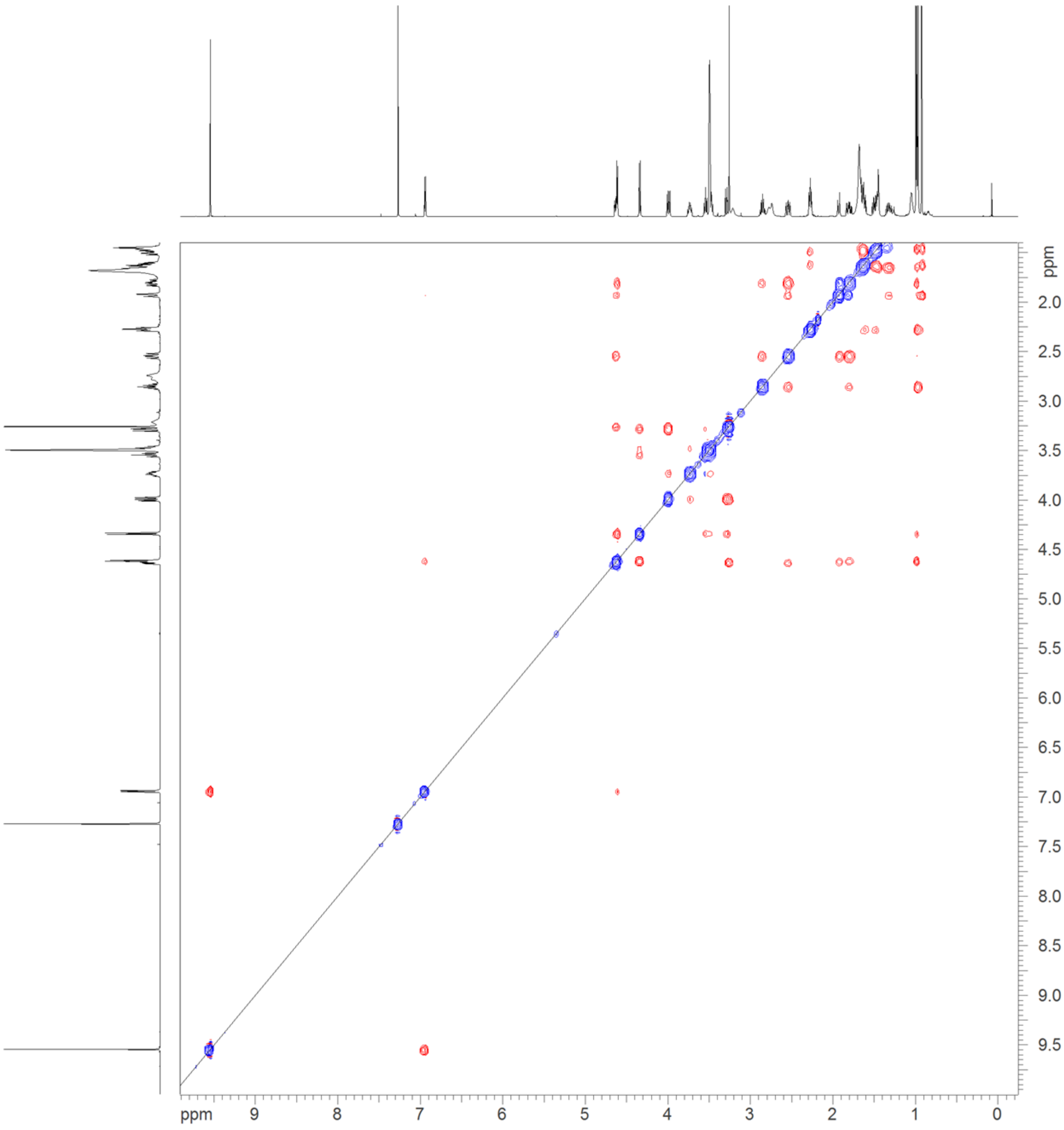

Figure S7: NOESY spectrum (500 MHz, $CDCl_3$) of Erinacine Z1 (1)

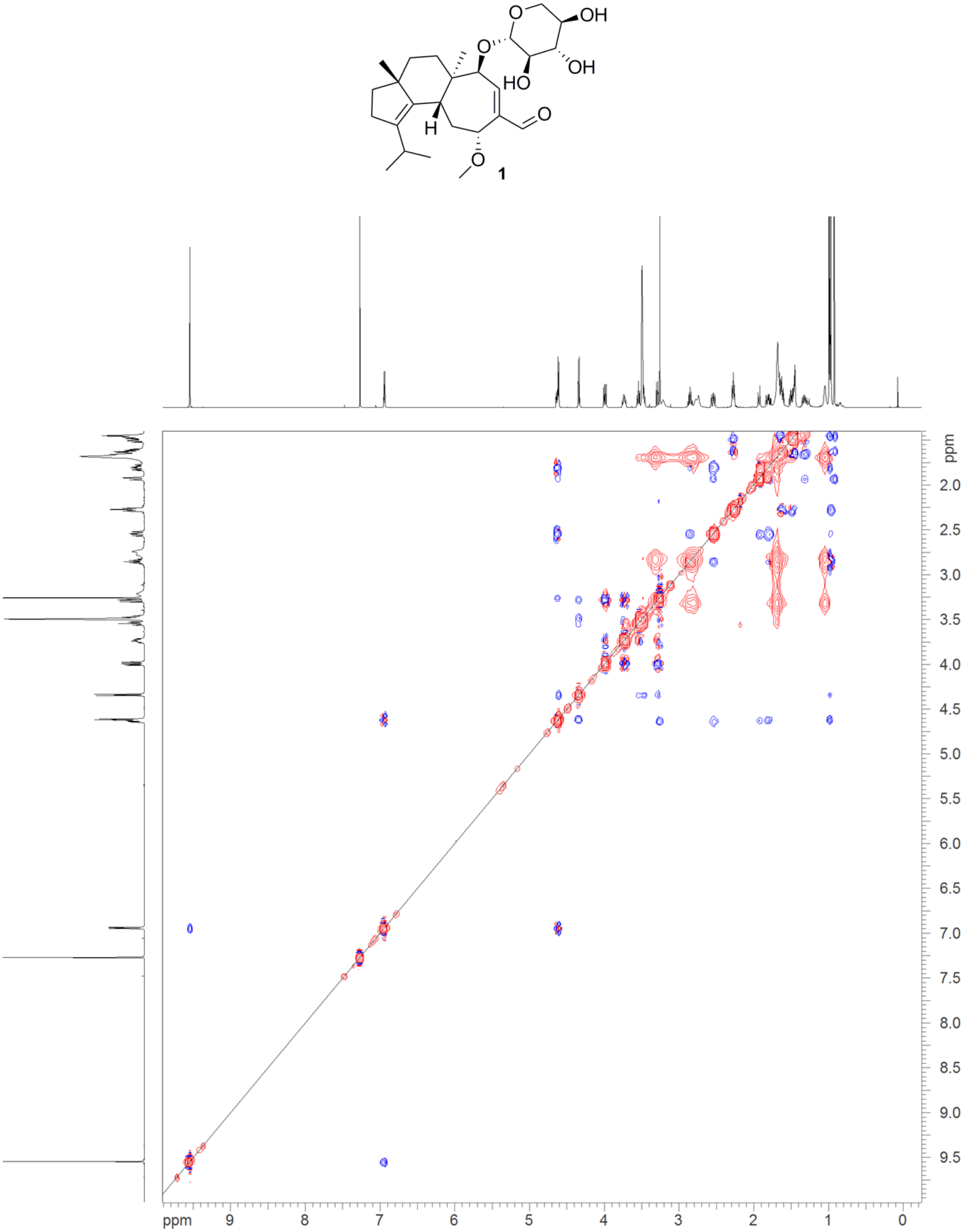

Figure S8: ¹H-NMR spectrum (700 MHz, acetone-d6) of Erinacine Z2 (2)

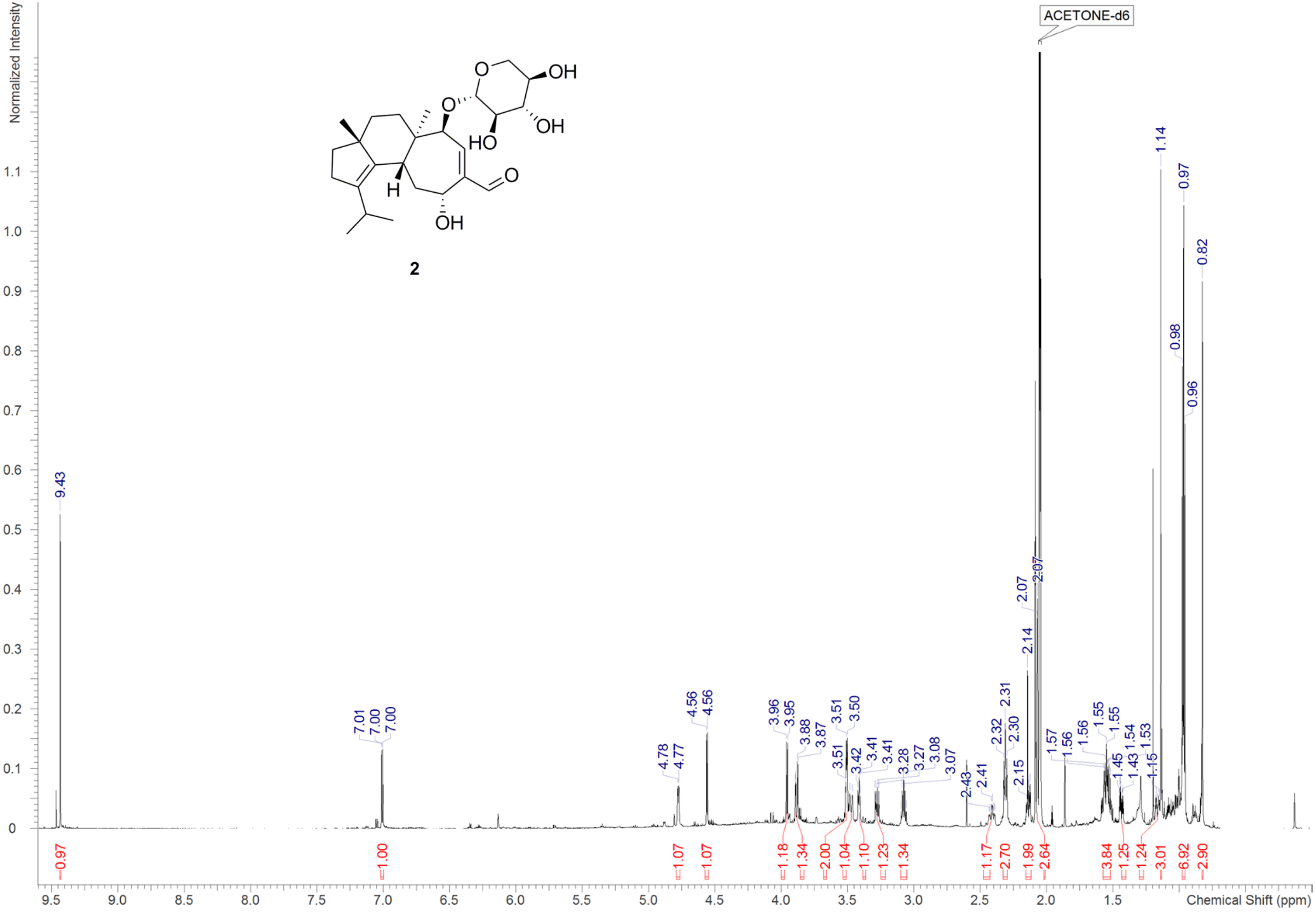

Figure S9: ^{13}C-NMR spectrum (700 MHz, acetone-d6) of Erinacine Z2 (2)

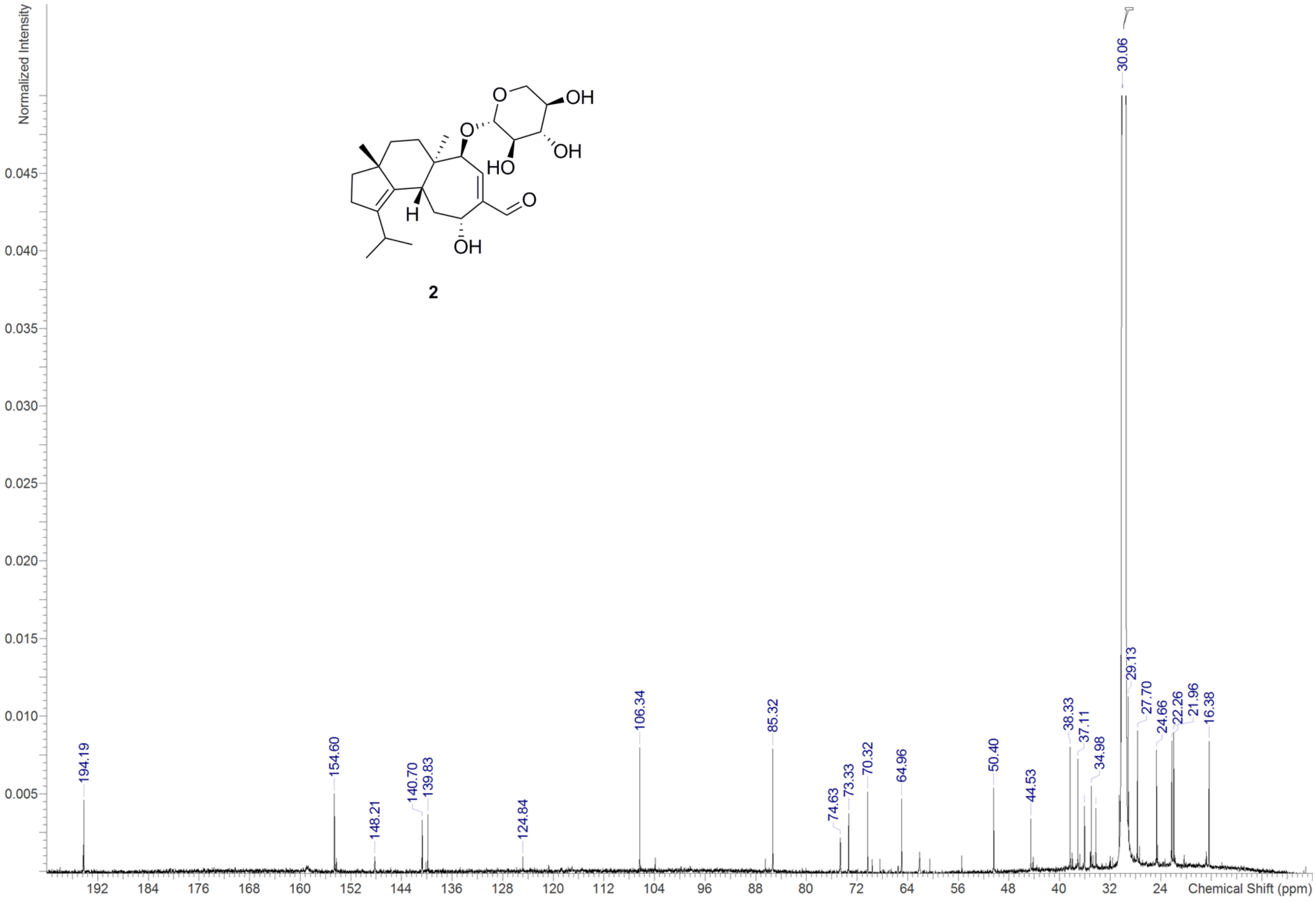

Figure S10: COSY-NMR spectrum (700 MHz, acetone-d6) of Erinacine Z2 (2)

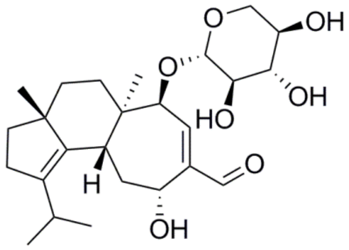

2

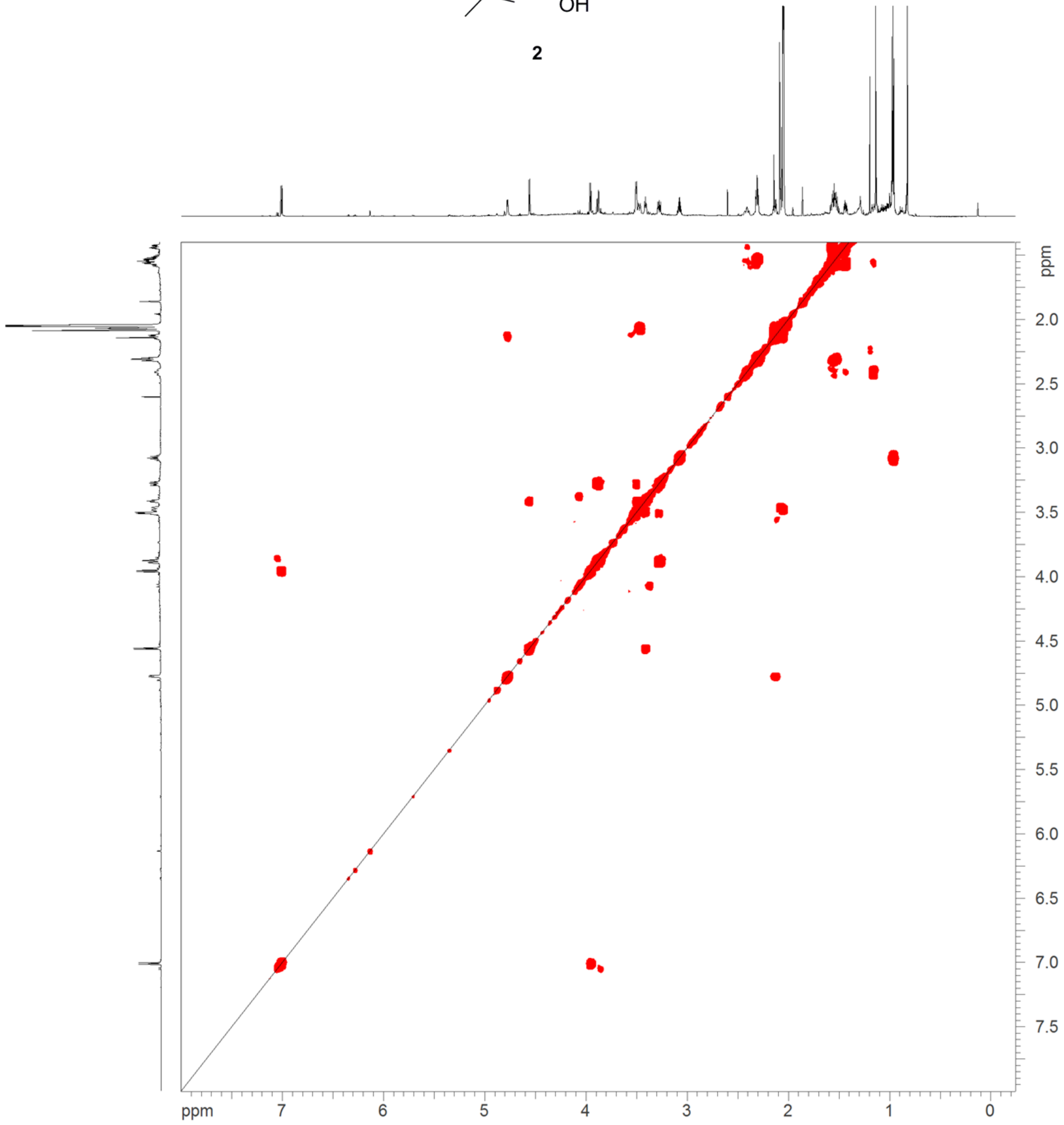

Figure S11: HSQC-NMR spectrum (700 MHz, acetone-d6) of Erinacine Z2 (2)

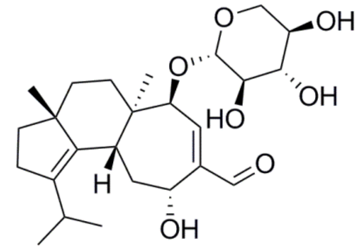

2

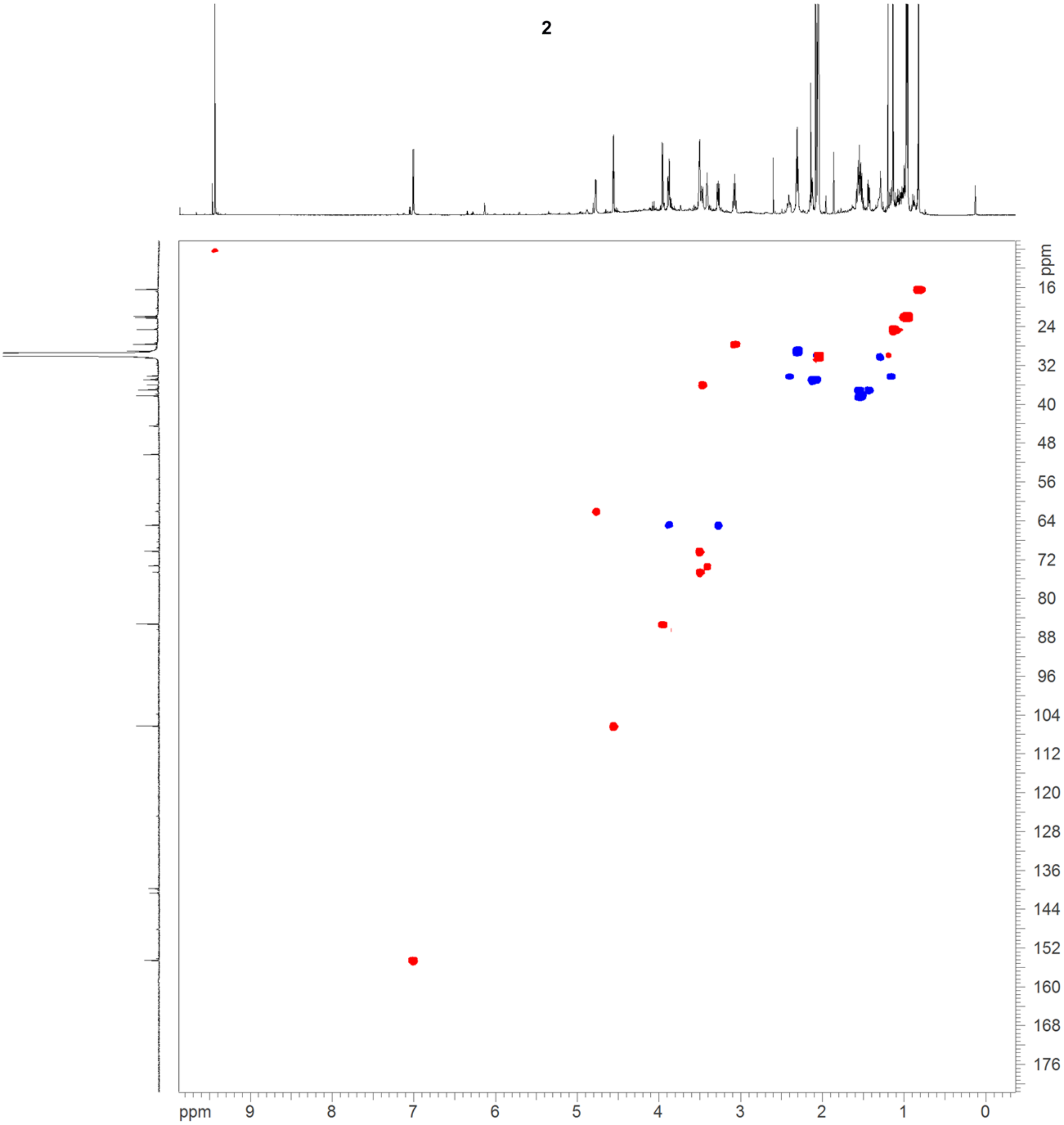

Figure S12: HMBC-NMR spectrum (700 MHz, acetone-d6) of Erinacine Z2 (2)

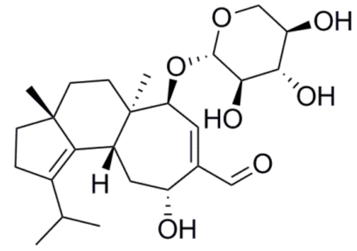

2

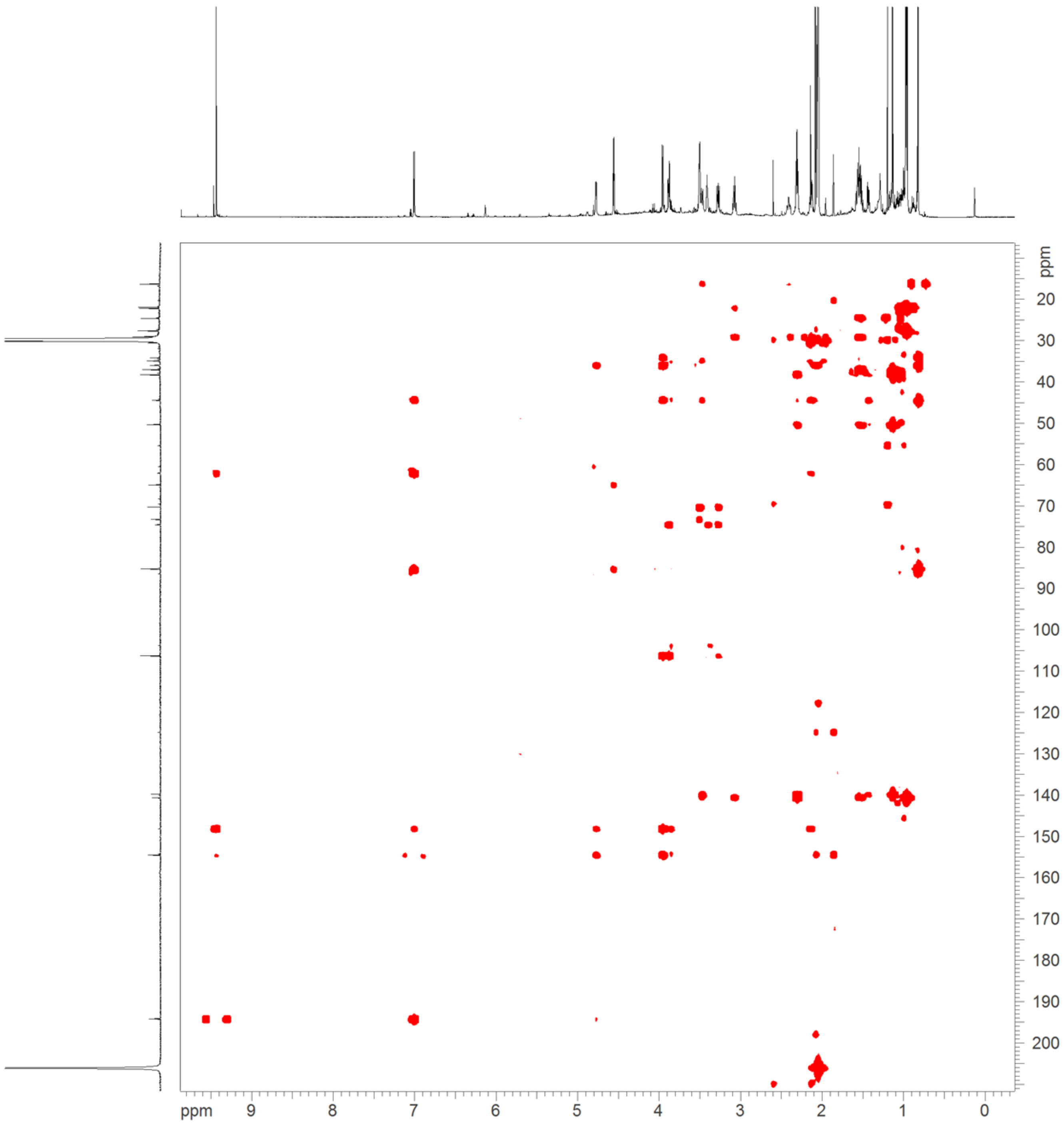

Figure S13: ROESY-NMR spectrum (700 MHz, acetone-d6) of Erinacine Z2 (2)

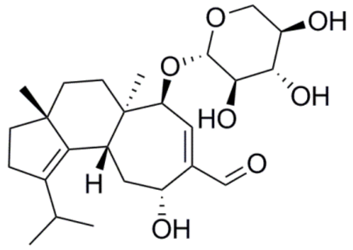

2

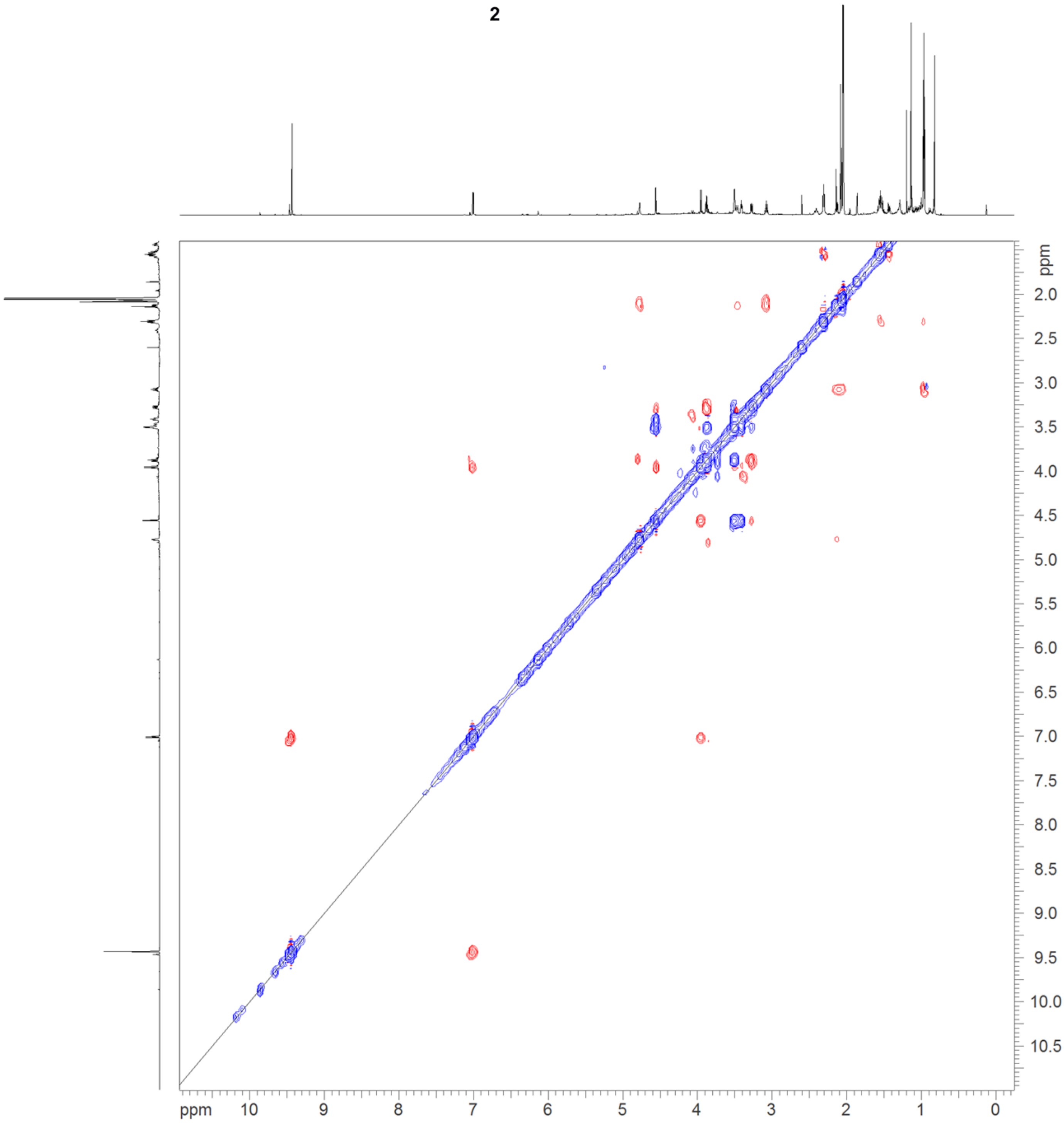

Supplement: Supplementary file 1 [file ijms-19-00740-s001.pdf]
